# Effectiveness of Ultrasonography Visual Biofeedback of the Diaphragm in Conjunction with Inspiratory Muscle Training on Muscle Thickness, Respiratory Pressures, Pain, Disability, Quality of Life and Pulmonary Function in Athletes with Non-Specific Low Back Pain: A Randomized Clinical Trial

**DOI:** 10.3390/jcm11154318

**Published:** 2022-07-25

**Authors:** Daniel Marugán-Rubio, J. L. Chicharro, Ricardo Becerro-de-Bengoa-Vallejo, Marta Elena Losa-Iglesias, David Rodríguez-Sanz, Davinia Vicente-Campos, Nerea Molina-Hernández, César Calvo-Lobo

**Affiliations:** 1 Faculty of Nursing, Physiotherapy and Podiatry, Universidad Complutense de Madrid, 28040 Madrid, Spain; danimaru@ucm.es (D.M.-R.); ribebeva@ucm.es (R.B.-d.-B.-V.); neremoli@ucm.es (N.M.-H.); cescalvo@ucm.es (C.C.-L.); 2 Department of Physiotherapy, Centro Superior de Estudios Universitarios La Salle, 28023 Madrid, Spain; 3 Grupo FEBIO, Universidad Complutense de Madrid, 28040 Madrid, Spain; jlopezch@ucm.es; 4 Faculty of Health Sciences, Universidad Rey Juan Carlos, 28922 Madrid, Spain; marta.losa@urjc.es; 5 Faculty of Health Sciences, Universidad Francisco de Vitoria, Pozuelo de Alarcón, 28223 Madrid, Spain; davinia.vicente@ufv.es

**Keywords:** biofeedback, breathing exercises, diaphragm, low back pain, ultrasonography

## Abstract

Diaphragmatic weakness and thickness reduction have been detected in athletes with lumbopelvic pain (LPP). Strength training of inspiratory muscles may be necessary for athletes with LPP. Inspiratory muscle training (IMT) and visual biofeedback by rehabilitative ultrasound imaging (RUSI) have been proposed as possible interventions. Here, we determine the effectiveness of visual biofeedback by RUSI with a proposed novel thoracic orthotic device to facilitate diaphragmatic contraction in conjunction with high-intensity IMT in athletes with non-specific LPP. A single-blinded, parallel-group, randomized clinical trial was performed (NCT04097873). Of 86 participants assessed for eligibility, 64 athletes with non-specific LPP (39 males and 25 females; mean age, 33.15 ± 7.79 years) were recruited, randomized, analyzed and received diaphragm visual biofeedback by RUSI in conjunction with high-intensity IMT (RUSI+IMT; n = 32) or isolated high-intensity IMT (IMT; n = 32) interventions for 8 weeks. Diaphragmatic thickness during normal breathing, maximum respiratory pressures, pain intensity, pressure pain threshold on lumbar musculature, disability by the Roland–Morris questionnaire, quality of life by the SF-12 questionnaire and spirometry respiratory parameters were assessed at baseline and after the 8-week intervention. There were significant differences (*p* = 0.015), within a medium effect size (Cohen’s *d* = 0.62) for the forced expiratory volume in 1-s (FEV_1_), which was increased in the RUSI+IMT intervention group relative to the IMT alone group. Adverse effects were not observed. The rest of the outcomes did not show significant differences (*p* > 0.05). Diaphragm visual biofeedback by RUSI with the proposed novel thoracic orthotic device in conjunction with high-intensity IMT improved lung function by increasing FEV_1_ in athletes with non-specific LPP.

## 1. Introduction

Lumbopelvic pain (LPP) is prevalent among athletes [1,2]. Higher disability, psychological conditions, and poor quality of life have been reported as common alterations presented under LPP [3,4,5,6]. Concretely, the point, year and life prevalence estimations reached 67%, 94% and 84%, respectively, in athletes who suffered from LPP [7]. It is estimated that LPP has an annual economic burden of 96 million USD [8]. In addition, more than 7000 EUR per person was stated as the direct economic burden of LPP [9]. A recent systematic review with meta-analysis indicated that several interventions were proposed for athletes with LPP, i.e., exercise approaches effective in reducing pain and improving function, although manual therapy interventions, such as massage or spinal manipulation, and biomechanical treatments presented insufficient evidence. Thus, high-quality randomized clinical trials were urgently claimed to establish new effective interventions for athletes suffering from LPP [10].

Indeed, men presented an early lumbopelvic movement pattern concerning women under LPP [11]. The presence of non-specific LPP in athletes generated functioning trunk stabilization alterations, leading to the loss of core deep muscles’ anticipatory activation [12]. Furthermore, the stabilizer muscles, such as the internal oblique, transversus abdominis, pelvic floor muscles, multifidus and diaphragm, comprise the core, playing a key role in supporting trunk motor control and the stability of athletes. Several devices, including magnetic resonance imaging, electromyography and ultrasound, were proposed as reliable and valid tools to measure static and dynamic muscle function in different conditions, using rehabilitative ultrasound imaging (RUSI) as a reliable, valid and non-invasive technique to evaluate the deep trunk stabilizers at rest and during muscle activity [13,14]. The RUSI technique presented several benefits for musculoskeletal soft tissue assessments, including joints, tendons, nerves and muscles [15,16]. Concretely, a recent systematic review with meta-analysis and meta-regression revealed that RUSI provided high reliability and validity measurements, especially in the lumbopelvic region, claiming promising findings for diagnostic and intervention evaluations in the physical therapy and rehabilitation fields [17].

Thus, the RUSI technique was used to evaluate static and dynamic core, deep muscles’ function in athletes, focusing on the abdominal wall [18,19,20], multifidus and lumbar muscles [21,22,23,24] and pelvic floor musculature [25]. Sports injury rehabilitation and prevention strategies commonly focused on RUSI visual biofeedback interventions for core muscles in athletes with LPP [21,22]. However, no scientific evidence was found regarding functional and morphological alterations of the diaphragm muscle during breathing in athletes with LPP. Among RUSI modalities, B-mode was shown as a reliable and valid modality to perform transcostal assessments regarding diaphragm morpho-functional evaluations during breathing [26]. In line with these findings, magnetic resonance imaging studies revealed a thinner diaphragm in conjunction with its reduced excursion during breathing activity, indicating motor control alterations of this muscle under LPP [27]. In addition, the LPP condition was linked to greater diaphragm fatigability [28], reduced excursion and a higher diaphragmatic dome position [29]. Nevertheless, ultrasonography tools were cheaper and more portable compared to MRI devices, providing clear reasons for the increased use of the RUSI technique in physical therapy and rehabilitation [13,14,17].

Furthermore, muscle stabilization of the low back region was improved by diaphragm training in athletes with LPP [30]. Indeed, athletes’ performance was highly influenced by respiratory patterns [31]. The pelvic floor and diaphragm are synergistic muscles involving transversus abdominis, maintaining and increasing intra-abdominal pressure during postural modifications [32]. Although the key role of the diaphragm has been investigated for more than 50 years, there is a lack of knowledge about the mechanisms of the diaphragm as a main low back stabilizer [29]. Recently, diaphragm muscle dysfunction (as a low back stabilizer) was claimed as a possible mechanism associated with LPP in athletes, and diaphragm training could play a key role in LPP recovery among athletes [33,34].

Athletes with chronic low back pain suffer from weaknesses in the activity of core muscles. Functional breathing alterations, including diaphragm muscle alterations as inspiratory muscle training (IMT), are considered an effective intervention to improve respiratory function, core muscles activity, postural sway and pain intensity in this population [35,36]. Specifically, high-intensity inspiratory muscle training (IMT) for 8 weeks improved proprioceptive signals during postural control, muscle strength and severity of recurrent, non-specific low back pain [28]. In addition, a 6-week training program for stabilization of the multifidus, transverse abdominis, and pelvic floor muscles using visual biofeedback by RUSI revealed an increase in the cross-sectional area of the multifidus muscles as well as a decrease in the pain of athletes suffering from LPP [22]. Nevertheless, there is a lack of knowledge about using ultrasound as visual biofeedback to improve diaphragmatic IMT in athletes with LPP. According to prior publications of our research group [33,37], the diaphragmatic thickness reduction evaluated by transcostal RUSI in athletes with LPP suggested that diaphragmatic reeducation could be a relevant intervention for the LPP prevention, performance and rehabilitation of athletes [33]. Furthermore, a novel thoracic orthotic device was proposed to improve ultrasound probe fixation by providing reliable and valid transcostal RUSI measurements for diaphragm thickness at relaxed breathing, decreasing measurement errors and allowing visual biofeedback for diaphragm reeducation in LPP athletes [37]. Thus, we hypothesized that visual biofeedback by RUSI could facilitate diaphragmatic contraction, improving the effectiveness of high-intensity IMT in athletes with non-specific LPP. Lastly, the purpose of the present study was to determine the effectiveness of visual biofeedback by RUSI with the proposed novel thoracic orthotic device to facilitate diaphragmatic contraction in conjunction with high-intensity IMT on diaphragmatic thickness during normal breathing (main aim), maximum respiratory pressures, pain intensity, pressure pain threshold on lumbar musculature, disability, quality of life and spirometry respiratory parameters in athletes who suffered from non-specific LPP.

## 2. Methods

### 2.1. Design, Ethics and Registry

A single-blinded, parallel-group, randomized clinical trial (blinded evaluator) was prospectively registered at ClinicalTrials.gov under identifier number NCT04097873 and carried out from 21 July 2020 to 13 June 2022 to determine the effectiveness of using visual biofeedback with the RUSI technique with the proposed novel thoracic orthotic device to facilitate diaphragmatic contraction combined with high-intensity inspiratory muscle self-training (RUSI+IMT) versus the isolated use of high-intensity inspiratory muscle self-training (IMT), determining diaphragmatic thickness during respiratory activity, diaphragm strength by maximum inspiratory pressures, pain intensity, pain pressure threshold (PPT) on the lumbar musculature, disability, quality of life and spirometric respiratory parameters in athletes with non-specific LPP, according to the Consolidated Standards of Reporting Trials (CONSORT-2010) criteria [38].

Ethical requirements for human experimentation and The Helsinki Declaration were respected [39]. The research was approved on 9 October 2019 by the San Carlos Clinical Hospital ethics committee from Madrid, Spain, with approval code 19/421-E_BS. All study subjects provided a signed informed consent form before the study began.

In addition, a holding device for the fixation of the ultrasound probe to the thoracic orthosis was previously registered in the Spanish Patent and Trademark Office with code U202000080 and publication number ES1245754 on 24 August 2020. This device reduced measurement errors of diaphragm thickness measurements evaluated by the transcostal RUSI technique compared to probe manual fixation measurements [33,37]. This research work was supported by the Madrid Government from Comunidad de Madrid, Spain, by the Multiannual Agreement within Complutense University according to the reference project code PR65/19-22348 in line with the Program to Stimulate Research for Young Doctors from the V Regional Programme of Research and Technological Innovation program (V PRICIT).

### 2.2. Sample Size Calculation

The sample size calculation for the proposed clinical trial was performed using the difference between two independent groups using the G*Power 3.1.9.2 program (G*Power©, University of Dusseldorf, Dusseldorf, Germany) [40]. The thickness difference of the left hemidiaphragm during inspiration was considered as the main outcome measurement, given that this measurement was associated with muscular alterations in the lumbopelvic region, since this hemidiaphragm presented a relevant postural function and a moderate effect size, with a Cohen’s *d* of 0.63 proposed as necessary to normalize the diaphragmatic thickness difference shown by athletes with LPP [33]. A one-tailed hypothesis, the α error probability of 0.05, power of 0.80 according to a 1-β probability error and N_2_/N_1_ randomization ratio N_2_/N_1_ of 1 were proposed. Thus, a sample size of 64 athletes with non-specific LPP was necessary, divided into two groups of 32 athletes in each intervention group. Considering 25% of possible losses to follow-up, 80 athletes with non-specific LPP were recruited for the total sample size (40 participants per group).

### 2.3. Study Sample

A total sample of 80 athletes with non-specific LPP was recruited by a simple random sampling procedure. The considered criteria for study inclusion were athletes suffering from bilateral non-specific LPP for at least 6 weeks; showing a pain distribution located from the popliteal fossa to the iliac crest, including bilaterally positive active straight leg raise (ASLR) test; being semiprofessional or amateur athletes (according to prior studies in this population showing diaphragm thickness differences [33] and concurrent validity for the use of the proposed thoracic orthosis device [37]) within a sports training program for at least 2 h and 1 day per week and playing one competition per week, performing a moderate or vigorous physical activity with a metabolic equivalent index of level II and III (i.e., above 600 METs/min/week, following the International Physical Activity Questionnaire (IPAQ)); and between 18 and 65 years old [33,37]. The considered criteria for study exclusion were congenital modifications of the lumbopelvic region, rheumatic disorders, neuromuscular pathologies, body mass index (BMI) greater than 31 kg/m^2^, respiratory conditions, neurological alterations, history of surgery, lower extremity musculoskeletal conditions (i.e., fractures, chronic ankle instability or sprains), skin conditions and inability to perform the study procedure. Lastly, reductions in physical activity or rest periods for more than 4 weeks and hyperventilation syndrome with a score of at least 24 points according to Nijmegen´s questionnaire were exclusion criteria for the present clinical trial [19,33,37].

### 2.4. Procedure, Randomization and Blinding

The singled-blinded, parallel-group, randomized clinical trial (blinded evaluator) was performed through a simple randomization sampling recruitment of 80 athletes with non-specific LPP who met the inclusion criteria, who were evaluated for descriptive data and outcome measurements and allocated to the two intervention groups (RUSI+IMT or IMT) according to a simple randomization process using the EPIDAT 4.1 program (Xunta de Galicia, Conselleria de Sanidade; Galicia, Spain). One group received isolated high-intensity inspiratory muscle self-training (IMT; n = 40) for 8 weeks [28]. The other group received the same IMT for 8 weeks plus ultrasound visual biofeedback (RUSI+IMT; n = 40) with the proposed thoracic orthotic device for diaphragmatic reeducation during normal breathing activity for 6 weeks [22,28,33,37]. An experienced physiotherapist applied both treatments in IMT and RUSI techniques. Outcome measurement assessments were carried out before interventions and after 8 weeks, coinciding with the end of both interventions by an experienced evaluator in the RUSI technique blinded to the treatment group allocation by numerical coding.

### 2.5. Interventions

**Figure 1:**
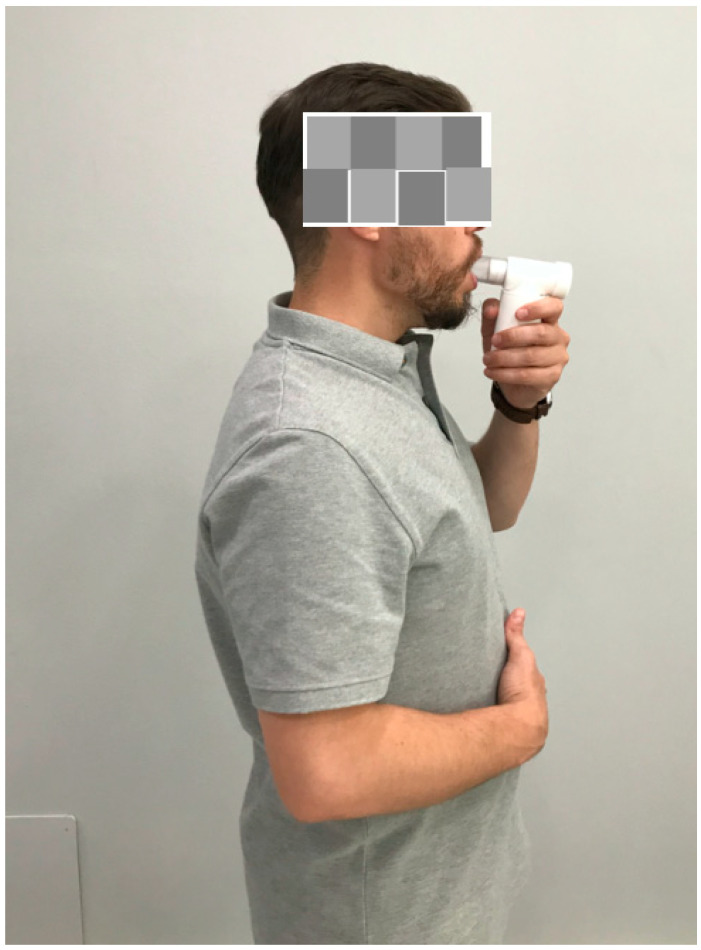
High-intensity inspiratory muscle self-training (IMT) by the POWERbreathe Medic.

The IMS group received isolated high-intensity inspiratory muscle self-training (n = 40) for 8 weeks, instructing athletes to breathe through a mouthpiece (POWERbreathe Medic; HaB International Ltd., Warwickshire, United Kingdom) with their nose occluded while standing, generating approximately a negative pressure corresponding to 60% of their maximum inspiratory pressure (MIP) via an inspiratory valve that resisted each inspiration (Figure 1). Patients were instructed to perform 30 breaths twice daily, 7 days per week, at a rate of 15 breaths per minute and a duty cycle of 0.5. In addition, all participants were trained to use primarily diaphragmatic breathing (“bucket-handle” motion) rather than thoracic breathing (“pump arm” motion) by providing verbal and tactile signals [28].

**Figure 2:**
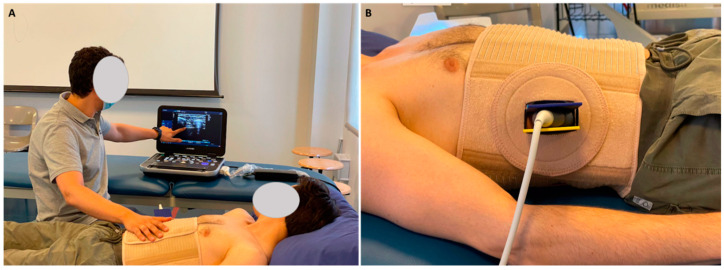
Ultrasound visual biofeedback by RUSI within the proposed orthosis device. (**A**) Ultrasound visual biofeedback by RUSI using the holding device to fix the ultrasound probe to the proposed orthosis device. (**B**) Adapter for fixation and support of the linear ultrasound probe placed perpendicular to the last intercostal space following the mid-axillary line.

The RUSI+IMT group received the same high-intensity IMT for 8 weeks plus ultrasound visual biofeedback by RUSI (n = 40) within the proposed thoracic orthotic device for diaphragmatic reeducation during normal breathing activity for 6 weeks (Figure 2). Patients were instructed in the same way for high-intensity IMT self-training in conjunction with diaphragmatic breathing reeducation using ultrasound visual biofeedback with the proposed thoracic orthosis to facilitate probe fixation and visualization of the ultrasound screen, selectively explaining diaphragmatic thickening during inspiration and correcting paradoxical breathing patterns [22,28,33,37]. Indeed, the holding device was performed to fix the ultrasound probe to the thoracic orthosis, decreasing RUSI measurement errors of the diaphragm thickness measurements and allowing for the transcostal visual biofeedback of the diaphragm muscle. The total thoracic mobility was always allowed using this device, and ultrasound gel was added in a space below the probe footprint, providing a complete visualization regarding the last intercostal space. The linear ultrasound probe was always placed perpendicular to the last intercostal space following the mid-axillary line, the patient was located at supine decubitus, and the probe was placed at the last intercostal space following Harper et al. [26]. This device was located on the right and left hemi-diaphragms at the last intercostal spaces by a bivalve adapter, which permitted the ultrasound probe holder insertion for fixation without interfering with the subjects’ breathing pattern and allowing total thoracic mobility according to correlations shown concerning measurement with the probe manual fixation procedure [37].

### 2.6. Descriptive Data

Sex was categorized into male or female, age was measured in years, height was measured in centimeters, weight was expressed in kilograms, BMI was determined as kg/cm^2^ following Quetelet´s index [41] and sport category was categorized into fitness or soccer to avoid the influence of this confounding variable, as well as the side of dominance. The dominance of the throwing-hand side and jumping foot were registered as right or left, and the smoking habit was categorized as yes or no [18,24,33,37]. According to the adequate psychometric properties of the International Physical Activity Questionnaire (IPAQ), the metabolic-equivalent-index-per-minute-per-week (METs/min/week) was determined to calculate physical activity and divided into moderate physical activity (from 600 to 1500 METs/min/week) or vigorous physical activity (equal or greater than 1500 METs/min/week) [42]. Lastly, Nijmegen’s test detailed the respiratory distress score due to its influence on diaphragm activity [24,33,37].

### 2.7. Primary Outcome: Ultrasound Measurements

All ultrasound measurements were performed by the thoracic orthosis device, fixing the ultrasound probe to determine bilateral diaphragm thickness outcomes during normal breathing using a randomized evaluation order considering both hemi-diaphragms. All ultrasound images were saved, coded and evaluated by a blinded examiner who performed diaphragm ultrasound thickness measurements of the coded ultrasound images by ImageJ software [33,37].

**Figure 3:**
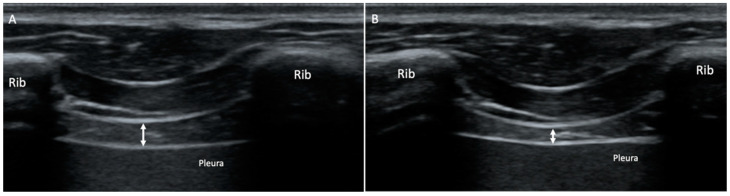
B-mode ultrasound imaging of the diaphragm thickness showing the last intercostal space at the mid-axillary line from the upper edge of the 12th rib to the lower edge of the 11th rib of the thorax. (**A**) Diaphragm thickness measurement marked by a white arrow at maximum inspiration (T^ins^) during normal breathing. (**B**) Diaphragm thickness measurement marked by a white arrow at maximum expiration (T^exp^) during normal breathing.

Transcostal RUSI measurements for both right and left hemidiaphragm thickness were performed in centimeters at maximum inspiration (T^ins^) and expiration (T^exp^), as well as their differences (T^ins-exp^) during normal breathing. A high-quality ultrasound device was used for all ultrasound images and measurements (Ecube-i7; Alpinion—Medical Systems; Seoul, Korea). These images were performed within a linear probe (L3_12T-type; 34-mm field-of-view; 128 elements), providing a frequency between 8-MHz and 12.0-MHz and a 45-mm footprint. Diaphragm thickness was measured at the supine position using B-mode ultrasonography using a prefixed pre-set of 3 cm deep, 12 MHz frequency, 64 points gain, 64 points dynamic range, and 1 focus placed at 2-cm depth [26,33,37]. All RUSI images were performed in grey-scale and converted in Digital Imaging and Communications in Medicine (DICOM) format, calibrated using the 2.0 v-ImageJ analysis software (U.S.-National Institutes of Health; Bethesda, MD, USA) and used to measure the thickness of both hemi-diaphragms [33,37]. In addition, the linear probe was located perpendicular to the last intercostal spaces according to the mid-axillary lines from the upper edge of the 12th rib to the lower edge of the 11th rib of the thorax, providing correct diaphragm visualization below the connective tissue of intercostal muscles during normal breathing (Figure 3). Three repeated measurements were performed to determine the diaphragm thickness of the right and left hemi-diaphragms at T^ins^, T^esp^ and T^ins-esp^, providing a total of three images regarding each parameter. Hemidiaphragm thickness measurements were performed by locating each electronic caliper inside both hyper-echogenic lines of the connective tissue around the diaphragm, performing thickness measurements at the intercostal space center. Three repeated measurements were used to calculate the mean. The probe fixation by the orthotic device reduced manual measurement errors and presented excellent reliability for ultrasound thickness measurements of the diaphragm muscle during normal breathing regarding intra-class correlation coefficients (ICC from 0.852 to 0.996), standard errors of measurement (SEM from 0.0002 to 0.054 cm) and minimum detectable changes (MDC from 0.002 to 0.072 cm), and avoiding systematic errors of measurement [37].

### 2.8. Secondary Outcomes

Secondary outcome measurements were maximum respiratory pressures, pain intensity, pressure pain threshold on lumbar musculature, disability, quality of life and spirometry respiratory parameters.

#### 2.8.1. Maximum Respiratory Pressures

Inspiratory and expiratory muscle strength was determined by maximum inspiratory (MIP) and expiratory (MEP) pressures, respectively, using the RP Check device (MD Diagnostics Ltd.; Chathman, United Kingdom) from the residual volume, according to the protocol proposed by the American Thoracic Society (ATS) and the European Respiratory Society (ERS) [43,44]. Maximum respiratory pressures were measured in cmH_2_O to compare both groups under absolute values. The measurement protocol was repeated at least three times or up to two reproducible efforts (within 5% for each other). One-minute intervals were applied between these measurements to avoid the fatigue of the respiratory musculature in the short term. The greatest of two reproducible values was considered for the analysis [34]. This procedure presented excellent inter-examiner reliability with an ICC from 0.914 to 0.925 [45].

#### 2.8.2. Pain Intensity

Pain intensity was measured by the Visual Analog Scale (VAS), considering the self-reported pain intensity average for the last week at rest, which consisted of a 100-mm horizontal line on which patients included the intensity of their pain, from “no pain” on the left side to the “worst pain imaginable” on the right side. This tool showed adequate reliability and validity within an ICC ranging from 0.65 to 0.88 with an adequate correlation of *r* of 0.74 with other pain scales [5,46].

#### 2.8.3. Pressure Pain Threshold

Pressure pain threshold (PPT) was measured from 0 to 10 kg/cm^2^ using a mechanical algometer (Wagner Instruments, Greenwich, CT). This device presented an ICC of 0.91, a coefficient of variation of 10.3%, a SEM of 0.19 kg/cm^2^ and a MDC of 0.54 kg/cm^2^. These coefficients indicated that this tool was a reliable, sensitive and reproducible instrument for evaluating the PPT in the center of the paravertebral spinal musculature bilaterally and perpendicularly to the spinous process of L3. PPT measurements were performed manually in a gradual manner until the patient began to feel pain. This process was carried out three times in the same place and within an interval from 30 to 60 s, using the mean of these three repeated measurements [23,47].

#### 2.8.4. Disability

Disability was determined by the Spanish version of the Roland–Morris Disability Questionnaire (RMDQ), which measured disability related to LPP, being a valid and reliable tool with an ICC of 0.87, comprising 24 items that measured the limitations of daily life secondary to LPP from 0 (“no disability”) to 24 points (“maximum disability”) [33,48].

#### 2.8.5. Quality of Life

Quality of life was measured by the Short-Form 12-item (SF-12) health questionnaire, which was applied to determine health-related, quality-of-life measure in the direct score and optimal normalized values to assess domains of physical and mental health as well as a total score, whose psychometric properties were valid and reliable with an α of Cronbach from 0.78 to 0.85 [49].

#### 2.8.6. Spirometry Respiratory Parameters

Spirometric parameters assessed airway airflow restrictions using the Datospir-600 Touch tool (e-20 software; SIBELMED; Barcelona, Spain). Spirometry respiratory values such as the forced expiratory volume during 1 s (FEV_1_; L), forced vital capacity (FVC; L), as well as FEV1/FVC ratio (%), were considered the most important parameters to reflect the airway disturbances at physiological level displayed. These values reflected lung function and were previously correlated with an *r* coefficient of 0.74 concerning chest wall expansion. These spirometry values presented good reliability within an ICC from 0.786 to 0.929 [50].

### 2.9. Statistical Analysis

The Statistical Package of Social Sciences (SPSS) by its 24.0 version (IBM; Armonk, NY, USA, IBM-Corp) was used for the statistical analysis, using an α error of 0.05 and a *p*-value < 0.05 as significant for a confidence interval (CI) of 95%. Analyses were performed considering two groups and the difference between two measurement moments (at baseline and at 8 weeks of intervention). The Kolmogorov–Smirnov test was used to analyze the normality of the distribution, as this test was recommended in health sciences for large enough sample sizes of more than 30 participants per group, and the Shapiro–Wilk test provided similar results [51]. All data were described as mean ± standard deviation (SD), upper and lower limits of the 95% CI and range (minimum-maximum). The student’s t-test compared differences between both groups for parametric data for independent samples using the *p*-value of the test according to Levene’s test for equality of variances (*p*-value ≥ 0.05 if there is equality of variances). Differences between both groups for non-parametric data were analyzed using the Mann–Whitney *U* test for independent samples. Categorical data were described by frequencies (n) and percentages (%). Analyses of differences in categorical data were compared using Fisher’s exact test to analyze dichotomous variables. In addition, the effect size of the outcome measurements’ differences between the two intervention groups was calculated using Cohen’s d with the formula $d=2t/\sqrt{gdl}$, and the effect size was categorized as very small effect size for *d* lower than 0.20, small effect size for *d* from 0.20 to 0.49, medium effect size for *d* from 0.50 to 0.79 and large effect size *d* equal of greater than 0.80 [33,52]. Lastly, a multivariate analysis by linear regression was performed to predict the differences in the outcome measurements that showed significant differences between both intervention groups according to the analyses described above (i.e., FEV_1_ differences). Linear regression analyses were performed using the “stepwise selection” method. The *R*^2^ coefficients were calculated to detail the quality of the adjustment, given that linear regression models only required at least a number of two subjects per variable for adequate estimation of the regression coefficients (*R*^2^) [53]. Descriptive and baseline data of the other outcome measurements were included in the linear regression analysis as independent variables, excluding from the analysis the baseline outcome measurement to be predicted (i.e., spirometry outcomes). The outcome measurement, which proved to be statistically significant between the study groups (i.e., FEV_1_ differences), was included in the linear regression analysis as a dependent variable. The pre-set probability *F* parameters were P_in_ of 0.05 and P_out_ of 0.10 [33].

## 3. Results

### 3.1. Flow Diagram

**Figure 4:**
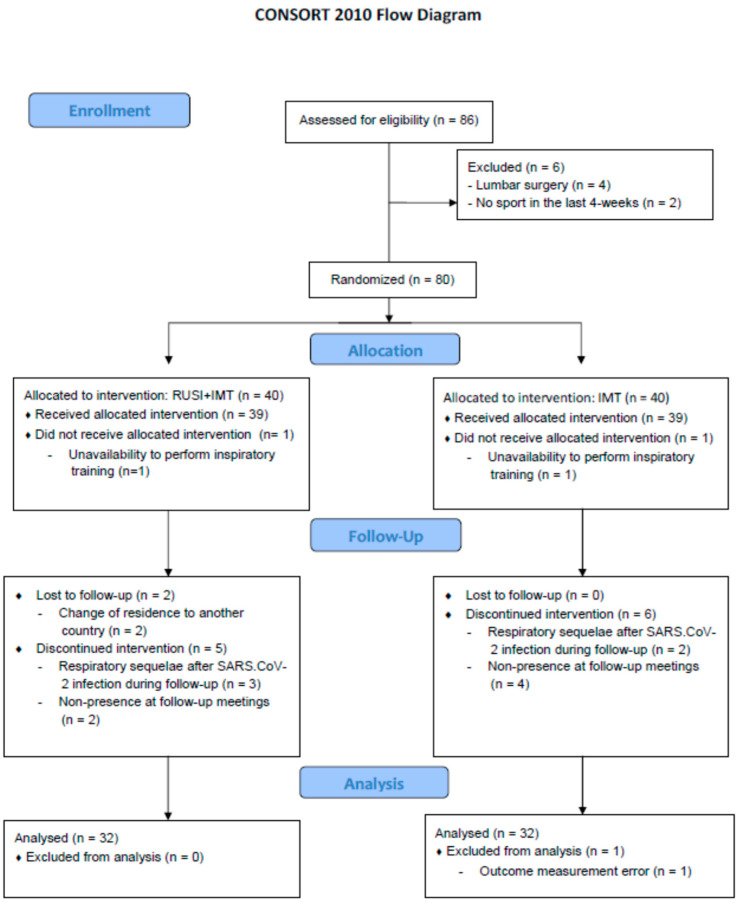
Flow diagram. Abbreviations: IMT, inspiratory muscle training; RUSI, Rehabilitative Ultrasound imaging.

Of 86 participants assessed for eligibility, six patients were excluded due to prior lumbar surgery (n = 4) and no sports performance in the last 4 weeks (n = 2). Thus, a total of 80 patients with LPP were randomized into RUSI+IMT (n = 40) and IMT (n = 40). In both groups, one patient did not receive the allocated intervention due to unavailability to perform inspiratory training, and the rest of the participants received the allocated intervention for both groups (n = 39). Regarding the RUSI+IMT group, two participants were lost to follow-up due to changes of residence to another country, and five patients showed discontinued interventions, three of them due to respiratory sequelae after SARS-CoV-2 infection during follow-up, two due to non-presence at follow-up meetings. Considering the IMT group, six patients presented discontinued interventions due to respiratory sequelae after SARS-CoV-2 infection during follow-up. In addition, one participant was excluded from analyses due to RUSI outcome measurement error. Finally, a total of 64 patients with LPP were analyzed per protocol (n = 32 in each group), as shown in Figure 4.

### 3.2. Descriptive Data

The total sample comprised 39 (60.94%) male and 25 (39.06%) female athletes with non-specific LPP. Both RUSI+IMT and IMT groups did not present statistically significant differences (*p* = 0.0305); the RUSI+IMT group included 22 (68.75%) male and 10 (31.25%) female athletes, and the IMT group comprised 17 (53.12%) men and 15 (46.88%) women. In addition, the main sports category carried out by both groups was fitness, with 29 (90.63%) athletes performing fitness and only three athletes (9.37%) performing soccer in each group (without significant differences between groups (*p* = 1.000)). Regarding differences in dominance between both groups, 28 (87.50%) athletes of the RUSI+IMT group and 30 (93.75%) of the IMT group presented a right side of dominance (*p* = 0.672), 27 (84.37%) participants showed right dominance of throwing-hand side in both groups (*p* = 1.000) and 27 (84.37%) athletes of the RUSI+IMT group and 30 (93.75%) of the IMT group reported right dominance of jumping foot (*p* = 0.672). Table 1 shows no significant differences (*p* > 0.05) for any descriptive data regarding age, weight, height, BMI, IPAQ and Nijmegen scores.

### 3.3. Baseline Outcome Measurements

Regarding the outcome measurements at baseline shown in Table 2, the sample was homogenous; there were no significant differences (*p* > 0.05) between both groups for RUSI diaphragm thickness for T^ins^, T^exp^ and T^ins-exp^ measurements of the right and left hemi-diaphragms, maximum respiratory pressures, pain intensity, bilateral paraspinal muscles PPT, disability, quality of life and spirometry respiratory parameters at baseline.

### 3.4. Comparison of Outcomes Differences

Regarding the comparisons for the difference variable (post–pre) of the primary outcome measurements between RUSI+IMT and IMT groups after 8 weeks, there were no significant differences (*p* > 0.05) within an effect size from very small to small (Cohen´s *d* = 0.00–0.46) for any bilaterally RUSI diaphragm thickness differences, as shown in Table 3. Considering the comparisons for the difference variable (post–pre) of the secondary outcome measurements between both intervention groups after 8 weeks, there were significant differences (*p* = 0.015) within a medium effect size (Cohen´s *d* = 0.62) for FEV_1_ increase in favor of the RUSI+IMT intervention concerning the isolated IMT intervention. The rest of the secondary outcome measurements, such as maximum respiratory pressures, pain intensity, bilateral paraspinal muscles PPT, disability, quality of life and the other spirometry respiratory parameters did not show any significant difference (*p* > 0.05) within an effect size from very small to small (Cohen´s *d* = 0.03–0.48).

### 3.5. Multivariate Linear Regression Analysis

A multivariate linear regression analysis was performed to predict the FEV_1_ differences between both groups after 8 weeks. A linear regression model (*R*^2^ = 0.307) predicted the FEV_1_ increase based on the left dominance of throwing-hand side (*R*^2^ = 0.156; β = 0.400; F_(1,62)_ = 11.485; *p* = 0.001), the RUSI+IMT intervention (*R*^2^ = 0.092; β = −0.285; F_(1,61)_ = 7.474; *p* = 0.008) and the higher baseline right hemi-diaphragm thickness difference at T^ins-exp^ (*R*^2^ = 0.058; β = 2.404; F_(1,60)_ = 5.058; *p* = 0.028).

## 4. Discussion

To our knowledge, this randomized clinical trial was the first study in the literature to determine the effectiveness of visual biofeedback by RUSI with the proposed novel thoracic orthotic device to facilitate diaphragmatic contraction in conjunction with high-intensity IMT in athletes who suffered from non-specific LPP [33,37].

Regarding the primary outcome measurements, adding RUSI visual biofeedback to IMT did not increase diaphragmatic thickness during normal breathing. This may be due to both groups receiving IMT, and the obtained diaphragm thickness differences were lower concerning control or sham interventions [34] and could have been influenced by measurement errors despite using the novel orthosis device [33,37].

Considering secondary outcome measurements, RUSI+IMT did not provide any improvement for maximum respiratory pressures, pain intensity, PPT on lumbar musculature, disability and quality of life concerning the isolated use of IMT. Some possible reasons to explain these issues may be that isolated high-intensity IMT provided notable clinical improvements in these outcomes, and low-intensity IMT could have shown the effects of RUSI visual biofeedback on diaphragm muscle more clearly [28]. Among spirometry respiratory parameters, the lung function was improved and predicted by an FEV_1_ increase in LPP athletes who received RUSI visual biofeedback in conjunction with high-intensity IMT, which could be linked to greater chest wall expansion [50]. Previously, the FEV_1_ reduction in LPP patients suggested an altered pulmonary function and core muscle dysfunction associated with LPP as a possible mechanism for this pulmonary dysfunction [54,55]. Based on these statements, we suggest that diaphragm visual biofeedback and IMT could improve pulmonary function in LPP athletes via core activation. Indeed, continuous physical activity performance in athletes may lead to adaptive changes in spirometry parameters, such as FEV_1_, and highlighted that there were necessary specific considerations of different respiratory patterns performed in different sport types [56].

Some possible reasons to explain the absence of effects under visual biofeedback by RUSI may be that this intervention was applied for 6 weeks following a prior study in other core muscles [22], whereas high-intensity IMT was applied for 8 weeks according to previous recommendations [28]. In addition, core muscles seemed to be activated simultaneously, which may be a possible explanation for the absence of effects under visual biofeedback due to simultaneous bilateral hemi-diaphragms, and the contraction of other core muscles for RUSI visual biofeedback could improve these results [32].

### 4.1. Future Studies

Future studies should evaluate the effects of RUSI visual biofeedback concerning a sham IMT intervention to assess changes in diaphragm thickness, maximum respiratory pressures, pain intensity and PPT on lumbar musculature, disability, quality of life and lung function in athletes with LPP. In addition, a simultaneous bilateral visual biofeedback for both hemi-diaphragms could provide a better core muscle co-activation and should be considered for future studies [33,37]. In addition, other conditions, such as heart failure [57], stroke [58] and lung alterations [59] have been greatly improved by IMT, and RUSI visual biofeedback by the proposed thoracic orthosis device could increase these beneficial effects. Finally, respiratory patterns of sports categories performed by athletes need to be further studied [56], especially under LPP and within the proposed orthosis by RUSI.

### 4.2. Limitations

The main limitation of the present study was the lack of control groups without intervention or within a sham or placebo device to evaluate the effects of RUSI diaphragm visual biofeedback in a more isolated way [34]. We only consider high-intensity IMT because this intervention was more effective than low-intensity IMT in patients with low back pain, and low-intensity IMT could be considered for future studies since RUSI visual biofeedback effects could be observed in a clearer manner [28]. Despite maximum respiratory pressures being measured in cmH_2_O to compare both groups under absolute values (thereby avoiding bias), using non-normalized values could lead to misinterpretation [43,44]. Finally, different sports categories were considered in the present study, and moderate or vigorous physical activity levels and sports modalities should be separately studied due to the different possible respiratory patterns [56]. Although pain intensity was measured, indicating that athletes self-reported their lumbopelvic pain intensity average for the last week at rest, we acknowledge that the moderate pain intensity mean showed in both groups could have been confound by pain intensity during physical activity, and this issue should be considered in future studies differentiating between pain intensity at rest and during physical activity [5,46].

## 5. Conclusions

Diaphragm visual biofeedback by RUSI with the proposed novel thoracic orthotic device in conjunction with high-intensity IMT improved lung function by increasing FEV_1_ in athletes who suffered from non-specific LPP. Nevertheless, adding this RUSI biofeedback to IMT did not alter diaphragmatic thickness, maximum respiratory pressures, pain intensity, PPT on lumbar musculature, disability or quality of life concerning the isolated use of IMT in LPP athletes.

## Figures and Tables

**Table 1:** Descriptive data for the total sample and their comparison for RUSI+IMT and IMT groups.

Descriptive Data	Total Sample (n = 64) Mean ± SD (95% CI) [Range]	RUSI+IMT (n = 32) Mean ± SD (95% CI) [Range]	IMT (n = 32) Mean ± SD (95% CI) [Range]	*p*-Value
Age (years)	33.15 ± 7.79	32.37 ± 8.29	33.93 ± 6.55	0.406 *
	(31.29–35.01)	(29.38–35.36)	(31.57–33.30)	
	[19.00–52.00]	[19.00–52.00]	[23.00–51.00]	
Height (cm)	160.79 ± 23.39	168.98 ± 31.76	172.40 ± 9.82	0.577 ^†^
	(164.85–176.53)	(157.53–180.44)	(168.86–175.94)	
	[159.00–196.00]	[159.00–196.00]	[157.00–193.00]	
Weight (kg)	68.90 ± 10.49	70.28 ± 10.45	67.53 ± 10.51	0.393 ^†^
	(66.28–71.52)	(66.51–74.05)	(67.73–71.32)	
	[50.00–94.00]	[53.00–94.00]	[50.00–87.00]	
BMI (kg/m^2^)	22.51 ± 2.94	23.09 ± 2.35	22.73 ± 2.74	0.571 *
	(22.27–23.54)	(22.24–23.94)	(29.38–35.36)	
	[18.69–29.21]	[19.88–28.65]	[18.69–29.21]	
IPAQ (METs/min/week)	2600.62 ± 1131.17	2775.00 ± 1328.50	2426.25 ± 879.03	0.427 ^†^
	(2318.06–2883.18)	(2296.02–3253.97)	(2109.32–2743.17)	
	[1200.00–5760.00]	[1449.00–5760.00]	[1200.00–5040.00]	
Nijmegen (score)	6.48 ± 5.56	6.61 ± 5.64	6.25 ± 5.57	0.701 ^†^
	(5.09–7.87)	(4.68–8.75)	(4.26–8.25)	
	[0.00–19.00]	[0.00–18.00]	[0.00–19.00]	

CI, confidence interval; BMI, body mass index; IMT, inspiratory muscle training; IPAQ, International Physical Activity Questionnaire; METs/min/week, metabolic-equivalent-index-per-minute-per-week; RUSI, Rehabilitative Ultrasound Imaging. *p* < 0.05 was considered significant for a 95% CI. * Student *t*-test for independent samples was used. ^†^ Mann–Whitney *U* test for independent samples was used.

**Table 2:** Baseline outcome measurements for the total sample and their comparison for RUSI+IMT and IMT groups.

Baseline Outcome Measurements	Total Sample (n = 64) Mean ± SD (95% CI) [Range]	RUSI+IMT (n = 32) Mean ± SD (95% CI) [Range]	IMT (n = 32) Mean ± SD (95% CI) [Range]	*p*-Value
T^ins^ right diaphragm thickness (cm)	0.19 ± 0.06	0.19 ± 0.07	0.18 ± 0.06	0.537 ^†^
	(0.17–0.20)	(0.17–0.22)	(0.16–0.20)	
	[0.08–0.39]	[0.11–0.39]	[0.08–0.33]	
T^exp^ right diaphragm thickness (cm)	0.13 ± 0.04	0.14 ± 0.04	0.12 ± 0.04	0.051 ^†^
	(0.12–0.14)	(0.12–0.16)	(0.10–0.13)	
	[0.06–0.25]	[0.07–0.24]	[0.06–0.25]	
T^ins-exp^ right diaphragm thickness (cm)	0.05 ± 0.04	0.05 ± 0.05	0.06 ± 0.03	0.510 *
	(0.04–0.06)	(0.03–0.07)	(0.04–0.07)	
	[−0.03–0.16]	[−0.03–0.16]	[−0.01–0.15]	
T^ins^ left diaphragm thickness (cm)	0.18 ± 0.05	0.19 ± 0.06	0.17 ± 0.05	0.159 ^†^
	(0.16–0.19)	(0.16–0.21)	(0.15–0.19)	
	[0.10–0.37]	[0.10–0.34]	[0.11–0.37]	
T^exp^ left diaphragm thickness (cm)	0.14 ± 0.04	0.15 ± 0.04	0.13 ± 0.04	0.127 *
	(0.13–0.15)	(0.13–0.16)	(0.11–0.14)	
	[0.07 –0.26]	[0.07–0.26]	[0.08–0.24]	
T^ins-exp^ left diaphragm thickness (cm)	0.03 ± 0.03	0.03 ± 0.03	0.03 ± 0.03	0.836 *
	(0.03–0.04)	(0.02–0.05)	(0.02–0.04)	
	[−0.02–0.15]	[−0.02–0.10]	[−0.01–0.15]	
MIP (cmH_2_O)	94.76 ± 29.82	94.87 ± 31.40	94.65 ± 28.66	0.977 *
	(87.31–102.21)	(83.55–103.19)	(84.32–104.99)	
	[34.00–168.00]	[34.00–168.00]	[47.00–157.00]	
MEP (cmH_2_O)	121.71 ± 28.39	128.50 ± 28.55	114.93 ± 26.98	0.055 *
	(114.62–128.81)	(118.20–138.79)	(105.20–124.66)	
	[69.00–183.00]	[69.00–183.00]	[72.00–180.00]	
VAS (score)	6.31 ± 1.36	6.40 ± 1.41	6.21 ± 1.33	0.635 ^†^
	(5.97–6.65)	(5.89–6.91)	(5.73–6.70)	
	[4.00–9.00]	[4.00–9.00]	[4.00–9.00]	
Paraspinal right PPT (kg/cm^2^)	5.72 ± 1.72	5.82 ± 1.64	4.96 ± 6.28	0.642 *
	(5.29–6.15)	(5.23–6.42)	(0.15–0.19)	
	[2.20–10.00]	[3.50–9.00]	[2.20–10.00]	
Paraspinal left PPT (kg/cm^2^)	5.78 ± 1.70	6.05 ± 1.64	5.52 ± 1.54	0.245 ^†^
	(5.36–6.21)	(5.46–6.64)	(2.10–10.00)	
	[2.10–10.00]	[3.80–10.00]	[0.08–0.24]	
RMDQ (score)	4.37 ± 4.40	4.78 ± 5.01	3.96 ± 3.73	0.465 ^†^
	(3.27–5.47)	(2.97–6.58)	(2.62–5.31)	
	[0.00–22.00]	[0.00–22.00]	[0.00–14.00]	
SF-12 Physical health (direct scores)	16.20 ± 2.68	16.68 ± 2.66	16.12 ± 2.70	0.837 ^†^
	(15.73–17.07)	(15.72–17.64)	(15.14–17.10)	
	[8.00–20.00]	[8.00–20.00]	[10.00–20.00]	
SF-12 Mental health (direct scores)	22.32 ± 3.75	22.03 ± 4.08	22.62 ± 3.44	0.532 ^†^
	(21.38–23.26)	(20.55–23.50)	(21.38–23.86)	
	[12.00–27.00]	[12.00–27.00]	[14.00–27.00]	
SF-12 Total score (direct scores)	38.98 ± 5.32	39.03 ± 5.56	38.93 ± 5.16	0.945 ^†^
	(37.65–40.31)	(37.02–41.03)	(37.07–40.79)	
	[20.00–46.00]	[20.00–46.00]	[27.00–45.00]	
SF-12 Physical health (optimal normalized values)	75.07 ± 19.11	76.40 ± 19.16	73.75 ± 19.27	0.553 ^†^
	(70.30–79.85)	(69.49–83.41)	(66.79–80.70)	
	[14.00–100.00]	[14.00–100.00]	[29.00–100.00]	
SF-12 Mental health (optimal normalized values)	78.34 ± 16.91	77.65 ± 17.71	79.03 ± 16.32	0.748 ^†^
	(74.11–82.56)	(71.26–84.04)	(73.14–84.91)	
	[29.00–100.00]	[29.00–100.00]	[38.00–100.00]	
SF-12 Total score (optimal normalized values)	77.18 ± 15.16	77.84 ± 15.87	77.03 ± 14.66	0.935 ^†^
	(73.40–80.97)	(71.81–83.06)	(71.74–82.31)	
	[23.00–97.00]	[23.00–97.00]	[43.00–94.00]	
FEV_1_ (L)	3.78 ± 0.84	3.86 ± 0.77	3.70 ± 0.91	0.458 *
	(3.57–4.00)	(3.58–4.14)	(3.37–4.03)	
	[2.21–5.93]	[2.60–5.93]	[2.21–5.54]	
FVC (L)	4.14 ± 1.52	4.26 ± 0.85	4.03 ± 4.91	0.311 *
	(3.92–4.36)	(3.95–4.57)	(3.70–4.36)	
	[2.45–6.78]	[2.75–6.78]	[2.45–5.80]	
FEV_1_/FVC (%)	90.72 ± 6.11	90.40 ± 6.36	91.04 ± 5.93	0.680 *
	(89.19–92.24)	(88.10–92.69)	(88.89–93.18)	
	[71.28–99.84]	[71.28–99.78]	[80.63–99.84]	

CI, confidence interval; FEV_1_, forced expiratory volume during 1 s; FVC, forced vital capacity; IMT, inspiratory muscle training; MEP, maximum expiratory pressures; MIP, maximum inspiratory pressures; PPT, pressure pain threshold; RMDQ, Roland–Morris Disability Questionnaire; RUSI, Rehabilitative Ultrasound Imaging; SF-12, Short-Form 12-item health questionnaire; T^ins^, maximum inspiration time; T^exp^, maximum expiration time; VAS, visual analog scale. *p* < 0.05 was considered significant for a 95% CI. * Student *t*-test for independent samples was used. ^†^ Mann–Whitney *U* test for independent samples was used.

**Table 3:** Comparisons for outcome measurements differences after 8 weeks between RUSI+IMT and IMT groups.

Outcome Measurement Differences After 8-Weeks	RUSI+IMT (n = 32) Mean ± SD (95% CI) [Range]	IMT (n = 32) Mean ± SD (95% CI) [Range]	Cohen’s *d*	*p*-Value
T^ins^ right diaphragm thickness (cm)	0.03 ± 0.08	0.01 ± 0.06	0.28	0.265 ^†^
	(−0.001–0.06)	(−0.005–0.04)		
	[−0.22–0.17]	[−0.19–0.12]		
T^exp^ right diaphragm thickness (cm)	0.008 ± 0.06	0.004 ± 0.04	0.07	0.287 ^†^
	(−0.01–0.03)	(−0.01–0.02)		
	[−0.11–0.22]	[−0.14–0.09]		
T^ins-exp^ right diaphragm thickness (cm)	0.02 ± 0.10	0.01 ± 0.05	0.12	0.143 ^†^
	(−0.01–0.05)	(−0.008–0.03)		
	[−0.45–0.17]	[−0.09–0.14]		
T^ins^ left diaphragm thickness (cm)	0.01 ± 0.07	0.01 ± 0.07	0.00	0.790 *
	(−0.01–0.04)	(−0.009–0.04)		
	[−0.13–0.20]	[−0.22–0.20]		
T^exp^ left diaphragm thickness (cm)	−0.02 ± 0.04	0.001 ± 0.05	0.46	0.056 *
	(−0.04–0.007)	(−0.01–0.02)		
	[−0.09–0.08]	[−0.15–0.15]		
T^ins-exp^ left diaphragm thickness (cm)	0.03 ± 0.05	0.01 ± 0.04	0.44	0.106 *
	(0.01–0.05)	(−0.0005–0.03)		
	[−0.07–0.12]	[−0.07–0.15]		
MIP (cmH_2_O)	24.03 ± 12.04	19.53 ± 16.53	0.31	0.218 *
	(19.68–28.37)	(13.56–25.49)		
	[1.00–55.00]	[−38.00–52.00]		
MEP (cmH_2_O)	21.00 ± 13.22	14.59 ± 17.61	0.41	0.105 *
	(16.23–25.76)	(8.24–20.94)		
	[−2.00–59.00]	[−42.00–66.00]		
VAS (score)	−3.75 ± 2.17	−3.81 ± 1.73	0.03	0.698 ^†^
	(−4.53–2.96)	(−4.43–3.18)		
	[−9.00–0.00]	[−8.00–0.00]		
Paraspinal right PPT (kg/cm^2^)	2.53 ± 1.39	2.11 ± 1.53	0.28	0.261 *
	(2.03–3.03)	(1.56–2.67)		
	[−0.20–6.00]	[−0.20–2.30]		
Paraspinal left PPT (kg/cm^2^)	2.51 ± 1.54	2.40 ± 2.01	0.06	0.909 ^†^
	(1.96–3.07)	(1.67–3.12)		
	[−0.60–5.60]	[−1.80–5.20]		
RMDQ (score)	−2.65 ± 3.26	−1.93 ± 1.88	0.27	0.685 ^†^
	(−3.83–1.47)	(−2.61–1.25)		
	[−12.00–0.00]	[−7.00–0.00]		
SF-12 Physical health (direct scores)	1.03 ± 1.57	0.87 ± 1.00	0.12	0.813 ^†^
	(0.46–1.59)	(0.51–1.23)		
	[0.00–7.00]	[−1.00–3.00]		
SF-12 Mental health (direct scores)	0.28 ± 1.05	0.15 ± 0.80	0.13	0.400 ^†^
	(−0.09–0.66)	(−0.13–0.44)		
	[−2.00–5.00]	[−1.00–4.00]		
SF-12 Total score (direct scores)	1.31 ± 2.26	1.03 ± 1.23	0.15	0.944 ^†^
	(0.49–2.12)	(0.58–1.47)		
	[0.00–12.00]	[−1.00–5.00]		
SF-12 Physical health (optimal normalized values)	7.37 ± 11.25	6.28 ± 7.21	0.11	0.770 ^†^
	(3.31–11.43)	(3.68–8.88)		
	[0.00–50.00]	[−7.00–21.00]		
SF-12 Mental health (optimal normalized values)	1.37 ± 4.91	0.71 ± 3.77	0.15	0.359 ^†^
	(−0.39–3.14)	(−0.64–2.08)		
	[−9.00–23.00]	[−5.00–19.00]		
SF-12 Total score (optimal normalized values)	3.59 ± 6.40	2.84 ± 3.48	0.14	0.828 ^†^
	(1.28–5.90)	(1.58–4.09)		
	[0.00–34.00]	[−3.00–15.00]		
FEV_1_ (L)	0.30 ± 0.44	0.04 ± 0.39	0.62	0.015 *
	(0.14–0.46)	(−0.10–0.18)		
	[−0.60–1.74]	[−1.48–0.99]		
FVC (L)	0.32 ± 0.43	0.13 ± 0.35	0.48	0.063 *
	(0.16–0.47)	(0.004–0.26)		
	[−0.59–1.23]	[−0.61–1.10]		
FEV_1_/FVC (%)	−0.64 ± 5.66	−0.38 ± 4.65	0.05	0.842 *
	(−2.68–1.39)	(−2.06–1.29)		
	[−14.25–13.89]	[−13.70–11.06]		

CI, confidence interval; FEV_1_, forced expiratory volume during 1 s; FVC, forced vital capacity; IMT, inspiratory muscle training; MEP, maximum expiratory pressures; MIP, maximum inspiratory pressures; PPT, pressure pain threshold; RMDQ, Roland–Morris Disability Questionnaire; RUSI, Rehabilitative Ultrasound Imaging; SF-12, Short-Form 12-item health questionnaire; T^ins^, maximum inspiration time; T^exp^, maximum expiration time; VAS, visual analog scale. *p* < 0.05 was considered as significant for a 95% CI (**in bold**). * Student *t*-test for independent samples was used. ^†^ Mann–Whitney *U* test for independent samples was used.

## Data Availability

Raw data will be available upon the corresponding author’s request.
